# RACIAL DISPARITIES IN 30-DAY READMISSION RATES AMONG MEDICARE PATIENTS DISCHARGED TO SKILLED NURSING FACILITIES

**DOI:** 10.1093/geroni/igz038.2871

**Published:** 2019-11-08

**Authors:** Maricruz Rivera-Hernandez, Maricruz Rivera-Hernandez, Momotazur Rahman, Vincent Mor, Amal N Trivedi

**Affiliations:** Brown University, Providence, Rhode Island, United States

## Abstract

The 30-Day All-Cause Readmission Measure is part of the Skilled Nursing Facility Value-Based Purchasing (SNFVBP) beginning 2019. The objective of the study was to characterize racial and ethnic disparities in 30-day rehospitalization rates from SNF among fee-for-service (FFS) and Medicare Advantage (MA) patients using the Minimum Data Set. The American Health Care Association risk-adjusted model was used. The primary independent variables were race/ethnicity and enrollment in FFS and MA. The sample included 1,813,963 patients from 15,412 SNFs across the US in 2015. Readmission rates were lower for whites. However, MA patients had readmission rates that were ~1 to 2 percentage points lower. In addition, we also found that African-Americans had higher readmission rates than whites, even when they received care within the same SNF. The inclusion of MA patients could change SNF penalties. Successful efforts to reduce rehospitalizations in SNF settings often require improving care coordination and care planning.

